# End-ischemic hypothermic oxygenated perfusion for extended criteria donors in liver transplantation: a multicenter, randomized controlled trial—HOPExt

**DOI:** 10.1186/s13063-023-07402-0

**Published:** 2023-06-06

**Authors:** Pierre Pradat, Solene Pantel, Marianne Maynard, Laure Lalande, Sylvie Thevenon, Rene Adam, Marc-Antoine Allard, Fabien Robin, Michel Rayar, Emmanuel Boleslawski, Olivier Scatton, Mircea Chirica, François Faitot, Philippe Bachellier, Olivier Soubrane, Kayvan Mohkam, Jean-Yves Mabrut, Mickaël Lesurtel

**Affiliations:** 1 Centre for Clinical Research, Hospices Civils de Lyon, Croix-Rousse University Hospital, Lyon, France; 2 Department of Pharmacy, Croix-Rousse University Hospital, Hospices Civils de Lyon, Lyon, France; 3 Department of HPB Surgery and Liver Transplantation, Paul Brousse University Hospital, Villejuif, France; 4 Department of HPB Surgery and Liver Transplantation, Pontchaillou University Hospital, Rennes, France; 5 Department of HPB Surgery and Liver Transplantation, Claude Huriez University Hospital, Lille, France; 6 Department of HPB Surgery and Liver Transplantation, Pitié Salpêtrière Hospital, Paris, France; 7 Department of HPB Surgery and Liver Transplantation, Michallon University Hospital, Grenoble, France; 8 Department of HPB Surgery and Liver Transplantation, Hautepierre University Hospital, Strasbourg, France; 9 Department of HPB Surgery and Liver Transplantation, Beaujon University Hospital, Clichy, France; 10 Department of Digestive Surgery and Liver Transplantation, Croix-Rousse University Hospital, Hospices Civils de Lyon, Lyon, France

**Keywords:** Liver transplantation, Extended criteria donors, End-ischemic hypothermic oxygenated perfusion

## Abstract

**Background:**

Given the scarce donor supply, an increasing number of so-called marginal or extended criteria donor (ECD) organs are used for liver transplantation. These ECD liver grafts are however known to be associated with a higher rate of early allograft dysfunction and primary non-function because of a greater vulnerability to ischemia–reperfusion injury. The end-ischemic hypothermic oxygenated machine perfusion (HOPE) technique may improve outcomes of liver transplantation with ECD grafts by decreasing reperfusion injury.

**Methods:**

HOPExt trial is a comparative open-label, multicenter, national, prospective, randomized, controlled study, in two parallel groups, using static cold storage, the gold standard procedure, as control. The trial will enroll adult patients on the transplant waiting list for liver failure or liver cirrhosis and/or liver malignancy requiring liver transplantation and receiving an ECD liver graft from a brain-dead donor. In the experimental group, ECD liver grafts will first undergo a classical static cold (4 °C) storage followed by a hypothermic oxygenated perfusion (HOPE) for a period of 1 to 4 h. The control group will consist of the classic static cold storage which is the gold standard procedure in liver transplantation. The primary objective of this trial is to study the efficacy of HOPE used before transplantation of ECD liver grafts from brain-dead donors in reducing postoperative early allograft dysfunction within the first 7 postoperative days compared to simple cold static storage.

**Discussion:**

We present in this protocol all study procedures in regard to the achievement of the HOPExt trial, to prevent biased analysis of trial outcomes and improve the transparency of the trial results. Enrollment of patients in the HOPExt trial has started on September 10, 2019, and is ongoing.

**Trial registration:**

ClinicalTrials.gov NCT03929523. Registered on April 29, 2019, before the start of inclusion.

**Supplementary Information:**

The online version contains supplementary material available at 10.1186/s13063-023-07402-0.

## Administrative information

Note: the numbers in curly brackets in this protocol refer to SPIRIT checklist item numbers. The order of the items has been modified to group similar items (see http://www.equator-network.org/reporting-guidelines/spirit-2013-statement-defining-standard-protocol-items-for-clinical-trials/).

**Table:** 

Title {1}	End-ischemic Hypothermic Oxygenated Perfusion for Extended Criteria Donors in Liver Transplantation – A multicenter, randomized controlled trial. HOPExt
Trial registration {2a and 2b}.	Registered on ClinicalTrials.gov under identifier NCT03929523 on April 29, 2019 before the start of inclusions. All items from the WHO Trial Registration Dataset can be found within the protocol.
Protocol version {3}	Protocol version 10, December 27, 2021
Funding {4}	French Ministry of Health: National Hospital Clinical Research Program 2018 (Programme Hospitalier de Recherche Clinique National 2018)
Author details {5a}	Pierre PRADAT^1^, Solène PANTEL^1^, Marianne MAYNARD^1^, Laure LALANDE^2^, Sylvie THEVENON^1^, René ADAM^3^, Marc-Antoine ALLARD^3^, Fabien ROBIN^4^, Michel RAYAR^4^, Emmanuel BOLESLAWSKI^5^, Olivier SCATTON^6^, Mircea CHIRICA^7^, François FAITOT^8^, Philippe BACHELLIER^8^, Olivier SOUBRANE^9^, Kayvan MOHKAM^10^, Jean-Yves MABRUT^10*^, Mickaël LESURTEL^10*^ ^1^ Hospices Civils de Lyon, Croix-Rousse University Hospital, Centre for Clinical Research, Lyon, France ^2^ Hospices Civils de Lyon, Croix-Rousse University Hospital, Department of Pharmacy, Lyon, France ^3^ Paul Brousse University Hospital, Department of HPB surgery and liver transplantation, Villejuif, France ^4^ Pontchaillou University Hospital, Department of HPB surgery and liver transplantation, Rennes, France ^5^ Claude Huriez University Hospital, Department of HPB surgery and liver transplantation, Lille, France ^6^ Pitié Salpêtrière Hospital, Department of HPB surgery and liver transplantation, Paris, France ^7^ Michallon University Hospital, Department of HPB surgery and liver transplantation, Grenoble, France ^8^ Hautepierre University Hospital, Department of HPB surgery and liver transplantation, Strasbourg, France ^9^ Beaujon University Hospital, Department of HPB surgery and liver transplantation, Clichy, France ^10^ Hospices Civils de Lyon, Croix-Rousse University Hospital, Department of Digestive Surgery and Liver Transplantation, Lyon, France
Name and contact information for the trial sponsor {5b}	Mr Alexandre Pachot Direction de la Recherche en Santé Hospices Civils de Lyon BP 2251, 3 Quai des Célestins, 69,229 LYON Cedex 02—France Tel: + 33 4 72 40 68 52.
Role of sponsor {5c}	The study sponsor and funder had no part in the study design, data collection, management, analysis, and interpretation of the data, writing of the report, and in the decision to submit the report for publication. They had no ultimate authority over any of these activities.

## Introduction

### Background and rationale {6a}

Ischemia–reperfusion (I/R) injury is universal in organ transplantation and results in microcirculatory and hepatocellular damage [1]. After liver transplantation, I/R leads to various degrees of graft dysfunction. Liver transplantation encompasses two forms of liver ischemia, namely cold and rewarming ischemia, both inducing hepatocellular injury. Cold ischemia occurs during organ retrieval, when the liver is cooled, perfused and then stored in a cold preservation solution. Rewarming ischemia is encountered during back-table graft preparation and also at the implementation stage while performing the vascular anastomoses before reperfusion [2]. Despite this fact, the preservation method for organ transplantation has been left unchanged for many years and simple static cold storage still remains the gold standard worldwide.

Given the scarce donor supply, an increasing number of so-called marginal or extended criteria donor (ECD) organs are used for liver transplantation, grafts which were previously rarely considered [3]. While there is no international consensus on the definition, ECD criteria include donor’s age over 65 years, donors in intensive care therapy for more than 7 days, obesity, fatty liver, prolonged cold ischemia time for over 12 h, and elevated liver enzymes or high natremia at any time in the donor. According to EuroTransplant and the French organ procurement organization (OPO) “Agence de la Biomédecine”, 40 to 50% of liver grafts are provided from ECD donors in Europe and in France, with the rate increasing over recent years (2016 report by the “Agence de la biomédecine”). These ECD liver grafts are however known to be associated with a higher rate of early allograft dysfunction (EAD) and primary non-function (PNF) because of a greater vulnerability to I/R injury. Although most authors agree that the graft function finally recovers, EAD is associated, in turn, with increased recipient susceptibility to sepsis [4], longer intensive care unit (ICU) and hospital stays [5–7], graft loss [8], and greater morbidity and mortality [9, 10].

Considering the excellent results achieved with machine perfusion preservation in kidney transplantation from ECD donors, machine perfusion techniques have been developed for liver grafts during the past decade and have shown promising results by decreasing reperfusion injury. Two major techniques for liver machine perfusion have been developed: normothermic or hypothermic oxygenated perfusion [11]. Normothermic liver perfusion with whole blood as perfusate requires an immediate start of normothermic perfusion at the place of organ procurement and during transport, as combination with previous cold storage has been shown to be disadvantageous [12]. Similarly, transportable cold perfusion devices for continuous cold perfusion after procurement require huge logistics and advanced technologies, not yet often available in routine clinical practice [13]. These continuous perfusion techniques have several drawbacks, including major logistical efforts and a risk of organ damage during perfusion and transport.

In contrast, the end-ischemic hypothermic oxygenated perfusion (HOPE) technique, which consists of using the machine perfusion procedure only during the last hours prior to transplantation, can be easily applied in the operating room where the transplant takes place, without any deviation from the usual organ transport and preparation methods used in the static cold storage procedure. Usually, during recipient hepatectomy, an interval of 1 to 3 h accumulates before the graft can be implanted. This period fits with the concept of a short-term hypothermic machine perfusion, applied after back-table preparation without delaying the transplant procedure. HOPE has been extensively tested in preclinical animal experimentations [14]. While the benefits of HOPE have been clinically demonstrated in donation after cardiac death (DCD) liver transplantations [15], there are sufficient arguments to hypothesize that the HOPE technique is superior to cold storage preservation of ECD liver grafts from brain-dead donors and may improve graft function and patient outcome after liver transplantation.

Despite the fact that liver machine perfusion is a promising method in liver transplantation, it has not spread into clinical practice as few results from randomized controlled trials (RCT) are currently available [16–18].

The HOPE technique appears to be simple and safe to perform because it is applied only a few hours before graft implantation in the transplantation center following usual transportation in static cold storage. Its feasibility and diffusion to teams are therefore easier.

### Objectives {7}

#### Primary objective

To assess the efficacy of HOPE, used before the transplantation of ECD liver grafts from brain-dead donors, in reducing postoperative early allograft dysfunction (EAD) within the first 7 postoperative days (POD) compared to simple cold static storage.

#### Secondary objectives

To assess the impact of HOPE on the following features compared to simple cold static storage:

- Quality of conservation
- Ischemia–reperfusion injuries
- Intra-operative events (bleeding, post-reperfusion syndrome)
- Postoperative outcomes (liver function tests not included in the definition of EAD, kidney function tests)
- 90-day morbidity and mortality
- Length of hospital stay and intermediate care unit stay
- Intra- and extrahepatic biliary complications within the first year after liver transplantation
- Mid-term outcome (3-month and 1-year patient survival and graft survival)
- Costs of liver transplantation with ECD graft using HOPE or not

To perform cost-effectiveness analysis.

## Trial design {8}

The HOPExt trial is a comparative open-label, multicenter, national, prospective, randomized, controlled, superiority study (1:1 ratio), in two parallel groups, with static cold storage as control.

## Methods: participants, interventions, and outcomes

### Study setting {9}

The study is conducted in eight transplantation centers located in University hospitals scattered throughout France. The list of participating centers is presented in Supplementary Material 1.

### Eligibility criteria {10}

Inclusion criteria

- Written informed consent prior to the performance of any study-specific procedure
- Affiliated to the French social security system
- Recipient age ≥ 18 years
- Patient undergoing primary liver transplantation
- Candidate for a first elective liver transplantation, whatever the indication
- Transplanted with a liver graft harvested from a brain-dead ECD defined as the presence of at least one of the following criteria:
  - Donor age > 65 years
  - Intensive care unit stay > 7 days
  - BMI > 30
  - Proven biopsy macro-steatosis ≥ 30%
  - Natremia > 155 mmol/L at any time
  - AST > 150 IU/L at any time
  - ALT > 170 IU/L at any time

Non-inclusion criteria

- Fulminant hepatic failure
- Retransplantation
- Split liver transplantation
- Living donor liver transplantation
- Graft donated after cardiac arrest (DCD graft)
- Domino transplantation
- Combined liver transplant
- Unexpected medical contraindication to liver transplantation
- Patient participating in other interventional research, excluding routine care research (old regulation) and category 2 research not interfering with primary endpoint analysis
- Patient under legal protection
- Patient deprived of liberty by a judicial or administrative decision
- Patient refusing to participate in the study
- Pregnant or lactating women
- Inability to understand information concerning the protocol

### Who will take informed consent? {26a}

Patients will be informed during pre-transplantation consultations with a surgeon or a hepatologist prior to being placed on the waiting list for liver transplantation. For patients already on the waiting list, information could be made during a follow-up outpatient consultation. A time to think will be left to the patient. Once the patient has asked all questions concerning the study and agrees to participate in the study, consent will be signed by both the patient and the investigator.

Final inclusion and randomization will be done after accepting a liver graft that meets the ECD criteria.

### Additional consent provisions for collection and use of participant data and biological specimens {26b}

No additional studies that may use the data collected in this trial are planned.

## Interventions

### Explanation for the choice of comparators {6b}

The standard French liver allocation rules will be followed. The study does not interfere or change the process of accepting or declining a liver graft offered to a patient in any way. If the liver is suitable for transplantation and all inclusion and exclusion criteria are met, the liver will be randomized to either HOPE after cold storage or static cold storage only. The control group will use the gold standard static cold storage technique (4 °C) with Institute George Lopez (IGL-1)® solution from graft harvesting until liver transplantation as routine practice in the eight centers.

### Intervention description {11a}

#### Study group

##### Identification of the medical device

ECD liver grafts will be perfused with hypothermic oxygenated perfusion (HOPE) via the portal vein only for a minimum of 1 h (ideally 1–4 h) after the “back-table” phase (graft preparation). This will be performed, in parallel with the recipient hepatectomy, using the CE-certified Liver Assist® perfusion pump/device (XVivo®, Sweden) with Machine Perfusion Solution (Belzer-MPS, CE-certified). Ideally, the graft should be placed in the machine before or at the time of the incision of the recipient to allow a duration of machine perfusion of the graft between 1 and 4 h. The machine perfusion time should not exceed 4 h. However, if the hepatectomy phase is longer than expected, the graft can remain on perfusion and used for transplantation. A notification of adverse events with specific interest will have to be done if the hypothermic oxygenated perfusion lasted more than 4 h.

The Liver Assist® is a pump system providing temperature-controlled dual oxygenated isolated perfusion of donor livers to bridge the time span between the donor hepatectomy and the liver transplantation in the recipient.

The Liver Assist® is a modular system consisting of five main modules:

- Portal vein pump unit (for portal vein perfusion)
- Hepatic artery pump unit (for hepatic artery perfusion) (*not used in this study* where only the portal vein is perfused)
- Thermo unit
- Trolley including table top
- Disposable set

The device incorporates neither medical substances nor non-viable materials of animal origin.

Date of CE mark: CE 663647 25/11/2016.

Market authorization: November 29, 2006.

#####  Intended use of the medical device

The Liver Assist is intended to be used for isolated ex vivo oxygenated machine perfusion of donor livers, for up to a period of 4 h.

##### Method of use of the device

If the patient is randomized into the machine perfusion group, the first part of graft preservation is similar to the control group. The graft is perfused and stored in Institute George Lopez (IGL-1®) solution at 4 °C during transport to the transplantation center.

As soon as the liver graft arrives at the transplantation center, the surgeon immediately prepares it on the back-table. The graft is then put on the Liver Assist perfusion machine around the time the transplant procedure begins.

Perfusion settings will be:

- Use of the same perfusion machine device in all centers (Liver Assist, Xvivo)
- Hypothermic perfusion (8–12 °C)
- Portal vein perfusion: portal vein is cannulated
- Adjusted perfusion flow: 150–250 ml/min under 3 mmHg in the portal vein
- Perfusate oxygenation at 70 kPA
- Use of 3 L of perfusion solution: Machine Perfusion Solution (Belzer-MPS, Bridge-To-Life, CE-certified)

##### Contraindications, warnings, possible risks

The Liver Assist perfusion machine is routinely used worldwide, and many experiments in patients have been reported in the literature [14]. There are no specific contraindications. The main advantage of hypothermic perfusion compared to normothermic perfusion, is that, in the event of a defect in the machine, the liver graft stays in hypothermic conditions and is protected against ischemic injuries. Even if the machine cannot be run, the liver graft is kept in static cold storage and can still be implanted in the recipient.

#### Control group

The control group will use the gold standard static cold storage technique (4 °C) with Institute George Lopez (IGL-1)® solution from graft harvesting until liver transplantation as routine practice in the eight centers.

### Criteria for discontinuing or modifying allocated interventions {11b}

In the following instances, patients will terminate the study prematurely if:

- Technical problem preventing any use of the perfusion machine
- Non-transplantable patients (e.g., peritoneal carcinosis)

However, all patients undergoing liver transplantation under the protocol conditions will be included in the modified intention-to-treat analysis (full analysis set).

### Strategies to improve adherence to interventions {11c}

The intervention is a single event requiring no adherence monitoring.

### Relevant concomitant care permitted or prohibited during the trial {11d}

There are no additional restrictions other than those listed in the non-inclusion criteria.

### Provisions for post-trial care {30}

The sponsor has subscribed to an insurance policy for the entire duration of the study, covering its own civil liability as well as that of all the physicians involved in the realization of the study. It will also insure the full compensation for harmful consequences of the research for the participating persons and their beneficiaries, except with evidence, at their responsibility, that the damage is not attributable to their mistake or to that of all consultants, without the possibility of being opposed to an act by a third party or the voluntary withdrawal of the person who had initially consented to participate in the research.

### Outcomes {12}

#### Primary endpoint

Difference between the two treatment arms in the proportion of patients with early allograft dysfunction (EAD). EAD (according to Olthoff et al. [7]) is defined by the presence of at least one of the following criteria:

- Bilirubin level > 10 mg/dL (i.e., 171 μmol/L) on postoperative day (POD) 7
- INR > 1.6 on POD 7
- AST or ALT level > 2000 IU/L within the first 7 PODs

Primary non-function (PNF) of the graft is the very last stage of any EAD and is defined by the presence of at least one of the following criteria:

- Graft loss within the first 7 PODs
- Patient’s death within the first 7 PODs

#### Secondary endpoints

1. Quality of conservation
  Dosage during perfusion by the HOPE machine (AST, ALT, LDH, hyaluronic acid, lactate levels in machine perfusate at 30 min and at the end of the perfusion)
  Additional 3 mL of machine perfusate will be sampled at 30 min and at the end of machine perfusion and stored at − 80 °C. Those samples will be used in case of future unexpected research about machine perfusate compounds
2. Difference between the two treatment arms in the proportion of patients with ischemia–reperfusion injuries
  Liver injury assessed by serum AST and ALT at 6 h (± 2 h), 12 h (± 2 h) and every day until POD7 after transplantation
  Post-reperfusion liver biopsy (histological changes, level of necrosis)
  Untargeted liver graft metabolic profiling (by high-resolution nuclear magnetic resonance—1H HR-NMR spectrometer) on liver biopsies on the back-table in both study groups and immediately after liver perfusion in the HOPE group. This test will be centralized in the Strasbourg center
3. Difference between the two treatment arms in the proportion of patients with intra-operative events
  Intra-operative blood transfusions (red blood cells, fresh frozen plasma, thrombocyte concentrate)
  Occurrence of post-reperfusion syndrome, defined as a 50% decrease in median arterial pressure during the 5 min following the graft revascularization
  Duration of surgery
4. Postoperative outcome (liver function tests not included in the definition of EAD)
  Difference in means between the two treatment arms for:
  Recipient blood level of factor V at 6 h (± 2 h), 12 h (± 2 h) and every day until POD7
  Arterial lactates at 6 h (± 2 h), 12 h (± 2 h) and every day until discharge from intensive care (and maximum until POD7)
  MEAF score (0–10) at POD3, including POD3 bilirubin, ALT max, and INR max at POD3. This score will be compared with the Olthoff’s definition of EAD and the L-GrAFT risk factor
  L-GrAFT risk factor (− 6 to + 6) including AST, INR, total bilirubin, and platelets every day until POD 10. This score will be compared with both MEAF score and Olthoff’s score
  Kidney function tests
    Daily serum creatinine level during the first 7 PODs
    Daily glomerular filtration rate (assessed using MDRD and CKD-EPI) during the first 7 PODs
    Difference between the two treatment arms in the proportion of patients requiring renal dialysis within the first 7 PODs
5. 90-day morbidity and mortality
  Difference between the two treatment arms in the proportion of patients with severe postoperative complication (defined as Dindo–Clavien classification ≥ 3) occurring before day 90 post surgery
  Difference in means between the two treatment arms of the comprehensive complication index (CCI) [19] at day 90
  Difference between the two treatment arms in the proportion of patients who were deceased before day 90 after surgery
6. Difference in means between the two treatment arms of the length of intermediate care unit stay and total hospital stay
7. Difference between the two treatment arms in the proportion of patients with intra- and extrahepatic biliary complications within the first year after liver transplantation, assessed by:
  Serum cholestasis parameter (bilirubin, gamma*-*glutamyl transferase, alkaline phosphatase) every 3 months until 1 year
  Liver contrast-enhanced MRI, including a magnetic resonance cholangiopancreatography (MRCP), 12 months after liver transplantation (except for patients who underwent a re-transplantation during the study)
8. Actuarial graft and patient’s survival rates at 3 months and 1 year
  Difference between the two treatment arms in the proportion of patients without graft loss at 3 months and 1 year.
  Difference between the two treatment arms in the proportion of patients alive at 3 months and 1 year.
9. Costs of liver transplantation with ECD grafts using HOPE or not, incremental cost-effectiveness ratio at 12 months post-surgery. Details about the cost-effectiveness analysis are given in Supplementary Material 2.

### Participant timeline {13}

#### Study calendar

Duration of the inclusion and randomization period: 36 months.

Duration of follow-up by patient: 12 months (± 30 days) after transplantation (36 months maximum).

Total duration of the study: 48 months (± 30 days).

Start of inclusions: September 2019.

#### Conduct of the study

The overall schedule and time commitment for trial participants are summarized in Table 1.

**Table 1:** Schedule of enrolment, interventions, and assessments in the HOPExt trial

Stages	V1 screening	V2 inclusion		V3	V4	V5–V11	V12	V13–V16
Time point	At inscription for LT or at pre-LT visit	D0 At hospitalization for LT	D0 HOPE group only	D0 + 6h (+/-2h) after LT	D0 + 12h (+/-2h) after LT	Day 1 to day 7 (+/-1day)	End of hospital stay	M3; M6: M9 and M12 (+/-30 days)
Actions								
Inclusion/non-inclusion criteria	X	X						
Informed consent	X							
Medical history and patient characteristics^1^	X							
MELD score	X At inscription	X						
Randomization^2^		X						
Clinical examination^3^		X					X	X
Biological analyses^4^		X (before LT)		X	X	X	X	X
Child–Pugh score		X (before LT)						
Donor characteristics^5^		X						
HOPE perfusion parameters^6^			X					
Bacteriological and fungal analyses^7^		X	X					
Intra-operative data^8^		X						
Liver biopsy^9^		X both groups	X					
Machine perfusate sample^10^			X					
ICU and hospital stay							X	
Morbidity (Clavien–Dindo score, CCI)							X	X At M3 only
Concomitant medication		X		X	X	X	X	X
Adverse events		X		X	X	X	X	X
Abdominal contrast enhanced MRI/MRCP								X At M12 only

^1^Patient characteristics: age, gender, height, weight, BMI, blood group, cause of cirrhosis, indication for transplantation, medical history (diabetes mellitus, arterial hypertension, transjugular intrahepatic portosystemic shunt), pretransplant status of residence (home, hospital ward or intensive care unit (ICU))

^2^Randomization will be performed after the harvesting team has macroscopically assessed the graft and confirmed that the graft will be harvested. After checking the inclusion and non-inclusion criteria, an online randomization tool will be used. Randomization will be stratified by center and MELD score at the time of transplantation with a cut-off of 30

^3^Clinical examination: vital signs (temperature, blood pressure, heart rate), body weight, height, BMI

^4^Biological analyses: AST, ALT, GGT, alkaline phosphatase, bilirubin, factor V, INR, platelets, creatinine, GFR, lactates. At V2 only, a pregnancy test (beta HCG) will be done for women of childbearing age

^5^Donor characteristics**:** age, gender, height, weight, BMI, blood group, length of stay in intensive care unit, cause of death, occurrence of cardiac arrest, biological test (AST, ALT, natremia)

^6^Parameters measured during HOPE perfusion (only in HOPE group): perfusion pressure, flow, temperature, duration of machine perfusion, perfusate oxygenation (partial pressure O2), and CO2 content (partial pressure CO2) at the beginning and at the end of machine perfusion; perfusate AST and ALT, LDH, hyaluronic acid, lactate levels at 30 min, and at the end of machine perfusion

^7^Bacteriological and fungal samplings will be taken on static storage solution (IGL-1) at the end of the back-table in both groups and of the perfusion solution at the end of machine perfusion for HOPE group

^8^Intra-operative data: surgical technique of transplantation (piggy-back vs. vena cava resection), length of procedure, transfusion needs (fresh frozen plasma, red blood cell, thrombocyte concentrate), occurrence of post-reperfusion syndrome (decrease of 50% of the median arterial pressure during the 5 min after the revascularization), cold ischemia time, circulatory support at the end of transplantation (noradrenaline (mg/h))

^9^A biopsy will be taken on the back-table before machine perfusion in both study groups, immediately after liver machine perfusion in the HOPE group, and after reperfusion of the liver in both groups

^10^3 ml sample will be taken from machine perfusion solution during HOPE perfusion (only in HOPE group), at 30 min and at the end of machine perfusion

#### Pre-screening

Patient eligible for liver transplantation after decision of the multidisciplinary board.

#### *Screening visit* = *V1*

All consecutive transplant candidates admitted to interdisciplinary transplant outpatient clinics requiring liver transplantation will be assessed for study eligibility by senior staff physicians (transplant surgeon). Patients on the waiting list for liver transplantation will be informed orally and in writing by a physician (transplant surgeon or hepatologist) about the study. Patients who are willing to participate, meet inclusion/non-inclusion criteria, and provide written informed consent will be included in the study.

Past medical history and patient characteristics will be collected: age, gender, height, weight, BMI, blood group, cause of cirrhosis, indication of transplantation, medical history (diabetes mellitus, arterial hypertension, transjugular intrahepatic portosystemic shunt), and pretransplant status of residence (home, hospital ward or ICU). The MELD score at inscription (INR, creatinine, bilirubin, and dialysis) is also recorded.

#### *Inclusion visit/randomization* = *V2*

#####  D0, visit 2

Verification that the consent form was signed by the patient and the investigator.

After the allocation and acceptance of the organs, the investigator will check the eligibility criteria. If the criteria are met, the liver recipient (study participant) will be randomized. The team of local investigators will be informed accordingly in order to set up the perfusion device. If the criteria are not respected, the patient will leave the study (premature termination of participation).

*Randomization*: it is important to note that absolutely no changes will be made to the national liver allocation rules. The study does not interfere or change the process of accepting or declining a liver offered to a certain patient in any way. Once a suitable recipient for the liver is identified, the recipient will be invited to come to the relevant transplant center for the surgical procedure as per routine procedure. Randomization will be performed after the allocation of the graft or after the harvesting team has macroscopically assessed the graft and confirmed that the graft will be harvested, according to the center practice. No study-related factors will be assessed before randomization. After checking the inclusion and non-inclusion criteria, an online randomization tool will be used. Randomization will be stratified by center and MELD score at the time of transplantation with a cut-off of 30.

*Prior to transplantation*: physical examination, vital signs, laboratory values (AST, ALT, GGT, alkaline phosphatase, bilirubin, factor V, INR, platelets, creatinine, GFR, lactates), MELD score (dialysis), CHILD–PUGH score (encephalopathy, ascites, albumin, prothrombin rate), and donor characteristics will be recorded. Concomitant medications and adverse events will be assessed.

*Liver transplantation*: back-table, recipient hepatectomy, and implantation will always be done by the principal investigator and sub-investigators. During recipient hepatectomy, the liver graft will be either stored in cold storage or perfused with the HOPE machine, according to the randomization. Parameters of the HOPE perfusion will be collected. Lot numbers of perfusate (Belzer-MPS) and perfusion device disposables will be recorded in the source document and eCRF.

In the control group, a biopsy of the liver graft will be taken at the beginning of the back-table preparation. Another biopsy will be taken after liver graft reperfusion in the recipient (routine practice).

In the experimental group, a biopsy of the liver graft will be taken at the beginning of the back-table preparation. Another biopsy will be taken at the end of the HOPE perfusion. A third biopsy will be taken after liver graft reperfusion in the recipient (routine practice). Study-related liver biopsies are (1) additional liver graft biopsy on the back-table in the control group and (2) additional liver graft biopsies before and after machine perfusion in the experimental group. Those biopsies are performed ex situ on the back-table without specific risk for the patient.

In order to look for any bacterial or fungal contamination during the whole process, samples of static storage solution (IGL in both groups) at the end of the back-table and samples of machine perfusion solution (Belzer-MPS) at the end of machine perfusion will be taken for bacteriological and fungal analyses. Those bacteriological and fungal samples will be analyzed by the microbiology laboratories of the participating centers in accordance with the European Pharmacopoeia 2.6.1 Sterility Analysis Protocol.

During surgery, intra-operative data are collected: surgical technique of transplantation (piggy-back vs. vena cava resection), length of procedure, transfusions needed (fresh frozen plasma, red blood cell, thrombocyte concentrate), occurrence of post-reperfusion syndrome (decrease of 50% of the median arterial pressure during the 5 min after the revascularization), cold ischemia time, and circulatory support at the end of transplantation (noradrenaline (mg/h)).

#### Follow-up visits

Visits 3–11 (6 and 12 h (± 2 h) after reperfusion and postoperative days 1–7, ± 1 day):

- Laboratory analyses (AST, ALT, GGT, alkaline phosphatase, bilirubin, factor V, INR, platelets, creatinine, GFR, lactates) will be performed. Concomitant medications and adverse events will be collected.

#### Visit 12 (last day of hospitalization)

Physical examination and laboratory analyses (AST, ALT, GGT, alkaline phosphatase, bilirubin, factor V, INR, platelets, creatinine, GFR, lactates) will be performed. Immunosuppression medication will be confirmed and post-transplant complications (Clavien–Dindo Score, CCI) will be assessed. Concomitant medications and adverse events will be collected. Post-transplant ICU and hospital stay will be assessed.

#### Visit 13 (3 months (± 30 days))

After liver transplantation, patients have regular checks at hospital, where physical examinations and lab test are performed (AST, ALT, GGT, alkaline phosphatase, bilirubin, factor V, INR, platelets, creatinine, GFR). During this appointment, the following will be recorded: the incidence of main postoperative complications (kidney function disorders, need for dialysis, infections, cholestasis and biliary obstructions, vascular complications, acute rejection, arterial or portal vein thrombosis, biliary fistula), Clavien–Dindo classification score, comprehensive complication index (CCI), reintervention, length of stay in intensive care unit, length of hospital stay, retransplantation, or death within 3 months after liver transplantation.

Adverse events and concomitant medications will be collected.

#### Visit 14–15 (6 months and 9 months after transplantation (± 30 days))

After liver transplantation, patients have regular checks at hospital, where physical examinations and lab tests are performed (AST, ALT, GGT, alkaline phosphatase, bilirubin, factor V, INR, platelets, creatinine, GFR). During this appointment, further examinations, modification of common medications, adverse events, or post-transplant complications will be noted and treated according to the routine management of patients after liver transplantation if necessary. Results of the checks will be documented in the eCRF.

Adverse events and concomitant medications will be collected.

#### Visit 16 (12 months after transplantation, outpatient control, final study visit closure, (± 30 days)

This is the last study visit. Patients will have physical examinations and lab tests (AST, ALT, GGT, alkaline phosphatase, bilirubin, factor V, INR, platelets, creatinine, GFR). During this appointment, further examinations, modification of concomitant medications, adverse events, or post-transplant complications will be noted and treated according to the routine management of patients after liver transplantation if necessary. Results of these checks will be documented in the eCRF. This is also the final control, including study closure and liver contrast enhanced MRI/MRCP.

Serious and adverse events will be evaluated throughout study participation.

### Sample size {14}

A sample size of 119 patients per randomized group (238 in total) is needed based on the following parameters:

- Expected decrease of EAD rate from 30% in the control group to 15% in the HOPE group (50% decrease of EAD based on previous preliminary clinical studies) [15, 20]
- Alpha: 0.05
- Power: 0.80
- Two-sided test

The sample size was calculated using the “pwr.2p.test” function of R (R Foundation for Statistical Computing, Vienna, Austria), package “Pwr”. In order to take into account a 10% proportion of dropouts or patients who prematurely terminate the study, 133 patients shall be recruited per group, i.e., 266 patients in total. Because there is about 20% of dropouts on the waiting list in France (2017 annual report of the “Agence de la Biomédecine”), and about 50% of liver grafts are provided from ECD, it is expected that 660 patients should be included in the study to be able to randomize 266 patients. In order not to slow down randomizations as they approach the total needed, inclusions will be possible beyond 660 patients within the limit of 1000 patients included.

According to EUROTRANSPLANT and the “Agence de la Biomédecine”, 50% of liver grafts are provided from ECD with the rate increasing over recent years. To be on the safe side, we expect that centers may randomize half of their potential transplanted patients with ECD, namely 25% of their total number of liver transplantations a year.

### Recruitment {15}

The 24-month inclusion period allows a reasonable mean rate of randomized patients of 1.39 patient/month/center. All participating centers are large and well-experienced liver transplantation centers in France. To ensure an adequate number of patients will be enrolled in the required time frame, the participating centers will be asked to report on a regular basis their problems related to enrolment, in order to find adequate responses to improve the enrolment rate. Moreover, to stimulate enrolment, a newsletter describing the enrolment status will be sent regularly to all centers.

## Assignment of interventions: allocation

### Sequence generation {16a}

Randomization will be performed after the allocation of the graft or after the harvesting team has macroscopically assessed the graft, according to the center practice.

It will be stratified by center and MELD score at the time of transplantation, with a cut-off of 30 (MELD < 30 versus MELD ≥ 30).

The randomization process will be centralized and carried out via Ennov Clinical software. The randomization list will be drawn up by the methodologist/biostatistician of the Clinical Research Center at the Croix-Rousse University hospital, Lyon, France.

The randomization list will be kept for 25 years in a tamper-proof envelope with the mandatory information (signature of the person in charge of the list, date and version number, complete title, and code of the research protocol). This list will also be sent to the Clinical Research Center data manager for implementation in the Ennov Clinical software.

The investigator or a person designated by the principal investigator will randomize patients via the Ennov Clinical software. Only authorized persons will be able to perform the randomization. A confirmation email will be sent to the person who performed the randomization after each randomization, to the investigator associated with the patient, and to the management center.

In case of cancellation of the transplant for any reasons, the data manager will cancel the randomization. The patient will remain included in the study for randomization in one of the study arms.

The whole process (randomization, modifications, etc.) will be recorded in the audit trail of the study, which will be integrated in the clinical data management system (CDMS). This audit trail will be transmitted at the end of the study to be archived with all the study documents.

### Concealment mechanism {16b}

Allocation concealment will be ensured via a central web-based system (Ennov Clinical® 7.5.720). The procedure to which a patient will be allocated will be disclosed only after enrolment in the study. Moreover, since a blocked randomization is used, the block size is not disclosed in the protocol making it impossible to predict the randomization sequence.

### Implementation {16c}

The allocation sequence will be generated by the biostatistician. Investigators at each study site will be responsible for patient enrolment in the study. Assignment of participants to each study group will be ensured by the central web-based system (Ennov Clinical® 7.5.720®) operated by local investigators, after verification of patient eligibility and inclusion in the study.

## Assignment of interventions: blinding

### Who will be blinded {17a}

Owing to the nature of the surgical procedure, it is not possible to blind the surgical and the anesthesiologist team for the group allocation. Because the patient cannot impact on the study outcomes after liver transplantation, there is no need to blind the patient. HOPExt is thus an open-label trial. However, in the event of isolated ALT or AST missing data for the primary endpoint, these data will be replaced by imputation on a case-by-case basis. The imputation will be performed blindly by the methodologist together with the principal investigator and will be validated by the DSMB. See the Methods in analysis to handle protocol non-adherence and any statistical methods to handle missing data {20c}. Moreover, for the analysis of the primary endpoint and some of the secondary endpoints, the data analyst will be masked to which group received the intervention (HOPE machine). A fixed code to denote each study group assignment (e.g., Group A; Group B) will be used instead.

### Procedure for unblinding if needed {17b}

Non-applicable since the HOPExt is an open-label trial.

## Data collection and management

### Plans for assessment and collection of outcomes {18a}

Investigators are responsible for the assessment and collection of outcomes, baseline, and other trial data. Data will be entered in the electronic case report form by delegated team members and will be monitored by trained clinical research assistants (CRA) designated by the sponsor. The digitalized version of the case report form is provided in Supplemental Material 2 (French version). CRA mandated by the sponsor will ensure the proper conduct of the study, collection of written data, their documentation, recording, and reporting in conformity with the Good Clinical Practices.

The investigator and the members of their team will accept to make themselves available during the quality control visits performed at regular intervals by the CRA. During these visits, the following elements may be reviewed:

- Informed consent
- Respect of the study protocol and the procedures defined therein
- Quality of the data recorded in the case report form: accuracy, missing data, consistency of the data with the source documents
- Management of the experimental treatments/strategies
- Declaration of serious adverse events

All visits will be the subject of a written monitoring report addressed to the investigator of the site visited and to the study coordinating structure.

### Plans to promote participant retention and complete follow-up {18b}

All follow-up visits of the HOPExt trial are part of the routine clinical follow-up after liver transplantation. No lost-to-follow-up are thus expected in this study.

### Data management {19}

Patient data needed for the study will be collected in an e-CRF. This e-CRF, developed via the Ennov Clinical software, will be specific to the study.

The patient code (see the paragraph “Confidentiality {27}”) will be the only information on the e-CRF which will link the data to the patient.

The e-CRF will only include the data needed to analyze the efficacy and safety of patients and for publication (information required by the protocol). Other data relating to the patient and necessary for their follow-up outside the study will be collected in their medical file. An explanation will be provided for each missing data.

At the end of the study, a paper print will be requested, which must be authenticated (dated and signed) by the investigator. A copy of the authenticated document for the sponsor must be archived by the investigator.

### Confidentiality {27}

In accordance with provisions concerning the confidentiality of data to which persons responsible for the quality control of a study involving human individuals have access (article L.1121–3 of the public health code), and in accordance with the provisions regarding the confidentiality of information relating, in particular, to the trial, the persons who participate, and the results obtained (article R.5121–13 of the public health code), the persons having direct access to the data will take all necessary precautions to ensure the confidentiality of the information related to the trials, to the persons participating and, in particular, with regard to their identity as well as the results obtained.

These persons, as with the investigators themselves, are subject to professional confidentiality (in accordance with the conditions defined by articles 226–13 and 226–14 of the penal code).

During the research involving human individuals or at its end, the data collected on the persons participating and sent to the sponsor by the investigators (or any other specialists) will be made anonymous.

Under no circumstances should the names or the addresses of persons concerned appear.

Only the first letter of the subjects’ surname and the first letter of their first name shall be recorded, accompanied by a coded number specific to the study indicating the center number and the inclusion order of the subject.

The sponsor will ensure that each person participating in the research has given their written agreement granting access to the individual data that concerns them and strictly necessary for the quality control of the study.

### Plans for collection, laboratory evaluation, and storage of biological specimens for genetic or molecular analysis in this trial/future use {33}

A metabolic assessment of the liver biopsies will be performed. Using pre-defined criteria (lactate > 8.4 and/or phosphocholine > 0.646), the level of MELG (metabolically extended liver grafts) both before and after machine perfusion will be compared within the experimental group. The level of MELG in both groups (experimental and control) will also be correlated with the risk of graft dysfunction or graft loss at 1 year.

In the control group, a biopsy of the liver graft will be taken at the beginning of the back-table preparation. Another biopsy will be taken after liver graft reperfusion in the recipient (routine practice).

In the experimental group, a biopsy of the liver graft will be taken at the beginning of the back-table preparation. Another biopsy will be taken at the end of the HOPE perfusion, and a third biopsy will be taken after liver graft reperfusion in the recipient (routine practice).

Immediately after collection (maximum 5 min after collection), the biopsy will be placed in 2-ml microvials to be either frozen in liquid nitrogen or put in allprotect®. They will then be stored at − 80 °C in each center.

Biopsies, realized at the beginning of the black-table for both groups and at the end of the HOPE perfusion for the experimental group, will be centralized at the end of the study at I-Cube laboratory (UMR 7357—Pr IJ Namer at l) in Strasbourg, France, for analysis. Approximately 30 metabolites of interest will be analyzed by spectrometry (Brucker Avance III 500 spectrometer). A histological study of the samples analyzed by spectroscopy will also be performed. The samples will be destroyed at the end of the analysis.

Since those biopsies will be performed on a liver graft from a deceased donor, an authorization from the Agence de la Biomédecine (ABM) was required and has been obtained.

## Statistical methods

### Statistical methods for primary and secondary outcomes {20a}

#### Analysis sets

##### Full analysis set (FAS)

All randomized and transplanted subjects will be considered for the FAS analysis (modified intention-to-treat analysis). This population will be used for efficacy analyses.

Patients randomized in the perfusion group (HOPE) and for whom perfusion cannot be initiated for any reason will be analyzed in the intention-to-treat analysis and considered in the group in which they were randomized. This population will be used for efficacy and feasibility analyses.

##### Per protocol (PP)

This population includes all subjects evaluable in FAS who ended the study without any major protocol deviation, analyzed in the group to which they are allocated by randomization. Major deviations will be defined individually during a data review/blind review meeting. Major deviations detected will be, for example:

- Non-respect of protocol timeframe for planned visits
- Randomization errors

This population will be used for efficacy analyses.

##### Safety population (SAF)

This dataset includes all transplanted subjects for whom at least one follow-up safety data is available. This population will be used for safety analyses.

The primary population of interest will be the FAS.

Descriptive summary statistics for continuous variables will include the number of observations (*n*), mean, standard deviation (SD), median, minimum (min), maximum (max), and 25th and 75th percentiles (IQR). Descriptive summary statistics for categorical data will include frequency counts and percentages *N* (%). Percentage calculations will be based on the number of patients for whom there are no missing data.

The primary endpoint will be expressed as the total number of cases in each group and in percentages, with a 95% confidence interval. The categorical variables of the secondary endpoints will also be expressed as the total number of cases in each group and in percentages with a 95% confidence interval.

Categorical variables will be compared between the two study arms using the chi-squared test or the Fisher’s exact test, as appropriate. The continuous variables of the secondary endpoints will be compared using the Mann–Whitney *U* test.

In order to take into account the potential effects of confounders on the primary endpoint, univariate and multivariate logistic regressions will be performed.

90-day morbidity and mortality will be studied using a Kaplan–Meier analysis and comparison between groups will be performed using the log-rank test.

A *p*-value < 0.05 will be considered as statistically significant.

### Interim analyses {21b}

Interim analysis will take place strictly for safety and ethical purposes. As soon as 20 patients per randomized group will be reached, data will be analyzed by the independent Data Safety Monitoring Board (DSMB). The trial will be stopped if the proportion of patients with major complications (Grade ≥ III) is statistically significantly higher (*p* < 0.001, Fisher’s exact test) in the HOPE group than in the control group. No statistical adjustment will be used in the interim analysis based on the sample size.

A second interim analysis will be conducted as soon as 50 per randomized group will be reached.

Results of these security analyses will be known by the DSMB members only.

### Methods for additional analyses (e.g., subgroup analyses) {20b}

There is no plan for any additional or subgroup analysis in this study.

### Methods in analysis to handle protocol non-adherence and any statistical methods to handle missing data {20c}

No missing data will be accepted for the primary endpoint and the main analysis will be based on patients with no missing data. In the event of isolated ALT or AST missing data for the primary endpoint, these data will be replaced by imputation on a case-by-case basis and this new dataset will be analyzed as a supplementary analysis. The imputation will be performed blindly by the methodologist together with the principal investigator and will be validated by the DSMB. In the event of missing or invalid data for secondary endpoints, the principal investigator and the person in charge of data analysis will decide whether these data should be considered as missing or replaced by imputation.

### Plans to give access to the full protocol, participant level-data, and statistical code {31c}

In conformity with the Good Clinical Practice:

- The sponsor is responsible for obtaining the agreement of all the parties implicated in the study in order to guarantee direct access to all the sites where the study will take place, to the source data, source documents, and reports, in the interests of quality control and audits by the sponsor;
- The investigators will provide the persons responsible for the follow-up, the quality control, or the audit of the study involving human individuals, the individual documents, and data that are strictly necessary for this control, in accordance with the current legal and regulatory provisions (article L1121-3 and R.5121–13 of the public health code).

The statistical code will be available upon request to the biostatistician in charge of data analysis.

## Oversight and monitoring

### Composition of the coordinating center and trial steering committee {5d}

The steering committee will be composed of the study coordinator (ML), the study methodologist (PP), the project manager (SP), the data manager (ST), the pharmacist (LL), and the manager of the Clinical Research Center of the Croix-Rousse Hospital (MM). The steering committee will be responsible for all aspects of the trial, including communication with investigators, updating the protocol and submitting amendments, and verifying compliance to study procedures. The steering committee will meet on a monthly basis or whenever necessary if any problem occurs.

### Composition of the data monitoring committee, its role, and reporting structure {21a}

A Data Safety Monitoring Board (DSMB) has been created for this study. The DSMB is an advisory committee responsible for helping the sponsor to proactively monitor and gauge patient safety and risk in the clinical trial. To this end, the DSMB reviews the data and any issues that may occur during the trial, in particular scientific, ethical and tolerance issues, which may modify the benefit/risk ratio. Following this review, the DSMB shall provide its recommendations in writing to the sponsor. These recommendations may concern in particular the continuation, modification, or termination of the study. The sponsor remains the decision maker of the measures to be implemented, following the recommendations of the DSMB. The modalities of the organization of this DSMB are described in a charter signed by the members of the DSMB at the beginning of the research. The DSMB includes at least two clinical experts and a methodologist/biostatistician.

Interim analysis will take place strictly for safety and ethical purposes. As soon as 20 patients per randomized group will be reached, data will be analyzed by the independent Data Safety Committee. The trial will be stopped if major complications (Grade ≥ III) are statistically significantly higher (*p* < 0.001, Fisher’s exact test) in the HOPE group when compared to the control group. A second interim analysis will be conducted as soon as 50 per randomized group will be reached.

### Adverse event reporting and harms {22}

#### Responsibilities of the investigator

All adverse events and incidents have to be investigated, reported and recorded, treated, and evaluated from consent signature until the end of the study and its resolution or stabilization.

All adverse events and incidents will be systematically reported in the adverse event reporting forms of the case report form (CRF). Each observed adverse event will be recorded individually. The intensity of the events will be graded according to the Clavien–Dindo classification.

All adverse events and incidents will be graded. If any adverse event develops and needs to be upgraded, a new adverse event should be added in the CRF (declaration of one adverse event per grade).

#### Reporting to the local correspondent for biovigilance

The investigator will report all incidents during collection of transplant to local correspondent of biovigilance according to the process established by the structure and to sponsor.

#### Reporting to the correspondent of materiovigilance

The investigator will report all serious and non-serious adverse reactions related to medical device to local correspondent of materiovigilance according to the process established by the structure.

All adverse events of severe intensity, life-threatening grade, and death (Grade 3 or above) shall be considered as *serious* (SAE) and must be notified to the sponsor without delay.

#### Serious adverse event (SAE) reporting

The investigator evaluates each adverse event in terms of its severity. The investigator shall notify the sponsor of all serious adverse events and serious incidents occurring during the trial (occurring during conservation of the organ and occurring in recipient), without delay and no later than 24 h from the day on which the investigator becomes aware of it, with the exception of those identified in the protocol as not requiring notification without delay. This initial notification shall be the subject of a written report and shall be followed by one or more additional detailed written report(s) within the 8 days following the first notification.

The investigator must document the event as well as possible (by means of copies of laboratory results or reports of examinations or hospitalizations, including relevant negative results, ensuring documents are anonymized and entering the patient's number and code), provide a medical diagnosis and establish a causal link between the serious adverse event, the medical device, and the procedure of implementation. The patient who has experienced an SAE must be followed up until complete resolution; stabilization at an acceptable threshold is achieved in the opinion of the investigator, or recovery to his previous state, even if the patient has been withdrawn from the trial.

#### Serious adverse events that do not require prompt notification to the sponsor (SAE form not sent to vigilant unit but collected in CRF)

During waiting list time (consent signature to transplantation), only death and serious events leading to withdrawal from the waiting list will be collected in the CRF.

#### Adverse events with specific interest

HOPE perfusion exceeding 4 h.

#### Causality assessment

The investigator must assess the causality of the SAE with the experimental treatment and/or the research procedures. The presence of confounding factors, like the concomitant treatments, the patient history, or other confounding factors must be taken into consideration. The causality is binary (reasonable related/not related).

#### Period of notification of SAE without delay to the sponsor by the investigator and procedures for monitoring serious adverse events

The investigator must notify the sponsor of the SAE and incidents without delay:

- From transplantation until the end of the patient involvement (M12 ± 30 days).
- Without any time limit for the severe adverse effect related to the experimental medical device, to the implementation procedure or to the research (for example: cancer, congenital malformation that occurs in the long term after exposure to the experimental device, etc.…).

#### Responsibilities of the sponsor

##### Declaration to the competent authorities

The sponsor assesses the causal relationship between the serious adverse event and the research. The sponsor shall report to the French Competent Authority (ANSM), any serious adverse reaction and serious incident, expected and unexpected that occurs in France and outside the national territory, during the research without delay. For reporting to the competent authority, SAE will be coded according to the MedDRA classification.

To ensure completeness of the safety data, the sponsor will work with local correspondent for biovigilance from the centers participating in the study.

An annual safety report will be submitted to the Ethic Committee and the Competent Authority (ANSM) by the sponsor within 60 days of the anniversary date of the study.

Any new issue related to the research and likely to undermine the safety of patients participating to the study will lead to urgent safety measures and information without delay by the sponsor to the competent authority and to the Ethic Committee.

### Frequency and plans for auditing trial conduct {23}

Trial audit, including audit of all enrolled participant data, will be performed by a dedicated auditing team designated by the study’s sponsor and independent from the study steering committee, investigators, and sponsor.

Trial audit will consist of verifying participants’ consent procedures and signed consent forms, verifying inclusion and exclusion criteria of enrolled participants, controlling the data collection of the primary outcome measure, controlling adverse event reporting, and reporting any major violation of study procedures. The auditing team will have full access to all required documents, including electronic medical records, in participating centers. Audit visits on the trial site will be performed per batch of 2 to 4 enrolled participants.

### Plans for communicating important protocol amendments to relevant parties (e.g., trial participants, ethical committees) {25}

In the event that a substantial modification is made to the protocol by the investigator, it will be approved by the sponsor. Before its implementation, the latter must obtain a favorable opinion from the ethics committee and an authorization from the ANSM within the scope of their respective competencies. A new consent will be collected from the people already participating in the study, if necessary.

## Dissemination plans {31a}

The trial is registered on ClinicalTrials.org under the reference number NCT03929523. The authors plan to submit the HOPExt final results to a peer-review journal within 12 months of the last patient’s end-of-participation date. The co-authors of the publication(s) will be the investigators and doctors involved, in proportion to their contribution to the study, as well as the biostatistician and the associated researchers.

The publication rules will follow the ICMJE guidelines and international recommendations [21].

Results of the trial will also be presented at national and international conferences.

## Discussion

The HOPE technique is currently under evaluation within 5 recent or ongoing RCTs conducted in Europe. Our team participated to the Zurich HOPE RCT which compared the HOPE technique to conventional static cold storage in all kinds of brain-dead donor grafts including ECD and normal grafts (NCT01317342) [22]. A significant difference of the primary endpoint (Clavien ≥ III complications) was not reached. Only liver-related Clavien ≥ IIIb complications occurred less frequently in the HOPE compared to the control group. Those mitigated results might be explained by the non-homogenous donor population including good liver grafts which may not have benefited from HOPE perfusion. The Groningen group (The Netherlands) conducted the DHOPE-DCD trial (NCT02584283) [16]. This trial assessed dual hypothermic oxygenated perfusion of 156 DCD liver grafts for the prevention of biliary complications after transplantation. Liver grafts were harvested from DCD donors and the HOPE technique was applied with dual perfusion via the portal vein and the hepatic artery of the graft. Arterial perfusion is more challenging than portal perfusion and may expose the liver graft to arterial vessel dissection, which can compromise the transplantation. In France, DCD liver transplantation (Maastricht III) belongs to a particular group of patients which benefits from a normothermic regional perfusion before organ procurement in order to maintain organ function [23]. Since these kinds of grafts are not concerned by any type of machine perfusion technology, no liver grafts from DCD donors will be included in the HOPExt trial. The German HOPE ECD-DBD trial (NCT03124641) is a multicenter RCT investigating the specific effects of HOPE on ECD organs from brain-dead donors [24]. While this is the same scope as the proposed HOPExt trial, the very unspecific primary endpoint (peak serum ALT) and the small sample size (23 patients per group) make difficult to draw definitive conclusions from the results. Finally, an Italian group has just reported the results of a similar RCT assessing the impact of HOPE in 110 patients who underwent liver transplantation with ECD-DBD organs (NCT03837197) [18]. The results are encouraging with a lower rate of EAD and a better 1-year graft survival in the HOPE group compared to the static cold storage group. However, the monocentric design of the study and the surprisingly low MELD score (median < 15) of the recipients may not reflect the actual conditions of a liver transplant program. Our multicenter trial will be stratified according to centers and MELD score of recipients avoiding bias in the selection of the patients.

The proposed RCT would be the first comparative clinical study on liver graft machine perfusion in France and would allow the French transplantation community to become familiar with liver machine perfusion. From an international point of view, it would be the first large RCT assessing the impact of HOPE on ECD from brain-dead donors and would be based on a robust clinical primary endpoint.

One limitation of the trial is the choice of definition of the EAD according to Olthoff [7]. Although it is not perfect because of its binary aspect, it allows the calculation of a sample size based on the results of previous trials. The use of the complication rate as the primary endpoint has been ruled out because of the usually high I/R non-specific morbidity after liver transplantation, which would hamper any conclusive results.

## Trial status

The HOPExt trial is currently enrolling patients. Enrolment of the first participant was on September 10, 2019. All eight participating centers are open and are currently enrolling patients. The trial expected date of conclusion is end of February 2023.

### Supplementary Information

**Additional file 1.** List of the investigators and centers participating in the HOPExt study.

**Additional file 2.** Health economic analysis.

**Additional file 3.** Informed consent form.

## Data Availability

After the publication of the trial’s results, the full study protocol, patient data, and statistical code will be made available upon reasonable request. Data access request will be reviewed by the HOPExt steering committee.

## Abbreviations

AE: Adverse event
ANSM: French National Agency for Medicine and Health Products Safety (Agence Nationale de Sécurité des Médicaments et des produits de Santé)
CRF: Case report form
DSMB: Data Safety Monitoring Board
EAD: Early allograft dysfunction
ECD: Extended criteria donor
HOPE: End-ischemic hypothermic oxygenated perfusion
MELD: Model for End-stage Liver Disease
SAE: Severe adverse event
