# Correction: End-ischemic hypothermic oxygenated perfusion for extended criteria donors in liver transplantation: a multicenter, randomized controlled trial—HOPExt

**DOI:** 10.1186/s13063-023-07471-1

**Published:** 2023-08-14

**Authors:** Pierre Pradat, Solene Pantel, Marianne Maynard, Laure Lalande, Sylvie Thevenon, Rene Adam, Marc-Antoine Allard, Fabien Robin, Michel Rayar, Emmanuel Boleslawski, Olivier Scatton, Mircea Chirica, François Faitot, Philippe Bachellier, Olivier Soubrane, Kayvan Mohkam, Jean-Yves Mabrut, Mickaël Lesurtel

**Affiliations:** 1 Centre for Clinical Research, Hospices Civils de Lyon, Croix-Rousse University Hospital, Lyon, France; 2 Department of Pharmacy, Croix-Rousse University Hospital, Hospices Civils de Lyon, Lyon, France; 3 Department of HPB Surgery and Liver Transplantation, Paul Brousse University Hospital, Villejuif, France; 4 Department of HPB Surgery and Liver Transplantation, Pontchaillou Univer- Sity Hospital, Rennes, France; 5 Department of HPB Surgery and Liver Transplan- Tation, Claude Huriez University Hospital, Lille, France; 6 Department of HPB Surgery and Liver Transplantation, Pitié Salpêtrière Hospital, Paris, France; 7 Department of HPB Surgery and Liver Transplantation, Michallon University Hospital, Grenoble, France; 8 Department of HPB Surgery and Liver Transplanta- Tion, Hautepierre University Hospital, Strasbourg, France; 9 Department of HPB Surgery and Liver Transplantation, Beaujon University Hospital, Clichy, France; 10 Department of Digestive Surgery and Liver Transplantation, Croix-Rousse University Hospital, Hospices Civils de Lyon, Lyon, France

**Correction: BMC Trials 24, 379 (2023)**

**https://doi.org/10.1186/s13063-023-07402-0**

Following publication of the original article [1], we have been informed that Table 1 has been misaligned during typesetting.

**Table 1:** Schedule of enrolment, interventions, and assessments in the HOPExt trial

***STAGES***	***V1*** ***Screening***	***V2*** ***Inclusion***			***V3***	***V4***	***V5 –V11***	***V12***	***V13-V16***
***Time point*** ***Actions***	***At inscription for LT or at pre LT visit***	***D0 At hospitalization for LT***	***D0*** ***HOPE group only***		***D0* + *6 h (*± *2 h) after LT***	***D0* + *12 h (*± *2 h)*** ***after LT***	***Day 1 to day 7 (*± *1 day)***	***End of hospital stay***	***M3; M6: M9 and M12 (*± *30 days)***
***Inclusion / non-inclusion criteria***	*X*	*X*							
***Informed consent***	*X*								
***Medical history and patient characteristics***^***1***^	*X*								
***MELD score***	*X At inscription*	*X*							
***Randomization***^***2***^		*X*							
***Clinical examination***^***3***^		*X*						*X*	*X*
***Biological analyses***^***4***^		*X* *(before LT)*			*X*	*X*	*X*	*X*	*X*
***CHILD–PUGH score***		*X* *(before LT)*							
***Donor characteristics***^*5*^		*X*							
***HOPE perfusion parameters***^*6*^			*X*						
***Bacteriological and fungal analyses***^***7***^		*X*	*X*						
***Intra-operative data***^*8*^		*X*							
***Liver Biopsy***^*9*^		*X* *both groups*	*X*						
***Machine perfusate sample***^***10***^			*X*						
*ICU and hospital stay*								*X*	
***Morbidity (Clavien-Dindo Score, CCI)***								*X*	*X* *At M3 only*
***Concomitant Medication***		*X*			*X*	*X*	*X*	*X*	*X*
***Adverse events***		*X*			*X*	*X*	*X*	*X*	*X*
***Abdominal contrast enhanced MRI/MRCP***									*X* *At M12 only*

^1^patient characteristics: age, gender, height, weight, BMI, blood group, cause of cirrhosis, indication for transplantation, medical history (diabetes mellitus, arterial hypertension, transjugular intrahepatic portosystemic shunt) pretransplant status of residence (home, hospital ward or intensive care unit (ICU))

^2^Randomization will be performed after the harvesting team has macroscopically assessed the graft and confirmed that the graft will be harvested. After checking the inclusion and non-inclusion criteria an online randomization tool will be used. Randomization will be stratified by center and MELD score at the time of transplantation with a cut-off of 30

^3^Clinical examination: vital signs (temperature, blood pressure, heart rate), body weight, height, BMI

^4^Biological analyses: AST, ALT, GGT, Alkaline Phosphatase, bilirubin, factor V, INR, platelets, creatinine, GFR, lactates

At V2 only, a pregnancy test (beta HCG) will be done for women of childbearing age

^5^Donor characteristics**:** age, gender, height, weight, BMI, blood group, length of stay in intensive care unit, cause of death, occurrence of cardiac arrest, biological test (AST, ALT, natremia)

^6^Parameters measured during HOPE perfusion (only in HOPE group): perfusion pressure, flow, temperature, duration of machine perfusion, perfusate oxygenation (partial pressure O2) and CO2 content (partial pressure CO2) at the beginning and at the end of machine perfusion; perfusate AST and ALT, LDH, hyaluronic acid, lactate levels at 30 minutes and at the end of machine perfusion

^7^Bacteriological and fungal samplings will be taken on static storage solution (IGL-1) at the end of the back-table in both groups, and of the perfusion solution at the end of machine perfusion for HOPE group

^8^Intra-operative data: surgical technique of transplantation (piggy-back vs. vena cava resection), length of procedure, transfusion needs (fresh frozen plasma, red blood cell, thrombocyte concentrate), occurrence of post-reperfusion syndrome (decrease of 50% of the median arterial pressure during the 5 minutes after the revascularisation), cold ischemia time, circulatory support at the end of transplantation (noradrenaline (mg/h))

^9^A biopsy will be taken on the back-table before machine perfusion in both study groups, immediately after liver machine perfusion in the HOPE group, and after reperfusion of the liver in both groups

^10^3ml sample will be taken from machine perfusion solution during HOPE perfusion (only in HOPE group), at 30 minutes and at the end of machine perfusion

The original article has been corrected.

## References

[1] Pradat Pierre (2023). End-ischemic hypothermic oxygenated perfusion for extended criteria donors in liver transplantation: a multicenter, randomized controlled trial—HOPExt. BMC Trials.

