# Supplementary material for: End-ischemic hypothermic oxygenated perfusion for extended criteria donors in liver transplantation: a multicenter, randomized controlled trial—HOPExt
Source: Trials. 2023 Jun 6;24:379. doi: 10.1186/s13063-023-07402-0 (PMC10243046; doi:10.1186/s13063-023-07402-0)
Supplement: Supplementary file 1 — Additional file 1. List of the investigators and centers participating in the HOPExt study. [file 13063_2023_7402_MOESM1_ESM.pdf]

List of the investigators and centers participating in the HOPExt study

Prof Mickaël LESURTEL
Department of HPB surgery and liver transplantation
Beaujon University Hospital
100, Bd Gl Leclerc
92110 Clichy, France
Phone: +33 1 40 87 50 00
Email : mickael.lesurtel@aphp.fr

Pr. Jean-Yves MABRUT
Department of Surgery and Liver Transplantation
Croix Rousse University Hospital
103, gde rue de la Croix Rousse
69317 LYON Cedex 04, France
Phone: +33 4 72 07 11 00 ; Fax: +33 4 72 07 29 27
Email : jean-yves.mabrut@chu-lyon.fr

Prof René ADAM
Department of HPB surgery and liver transplantation
Paul Brousse University Hospital
12 - 14 avenue Paul Vaillant Couturier
94804 Villejuif, France
Phone: +33 1 45 59 30 49
rene.adam@aphp.fr

Dr Michel Rayar
Department of HPB surgery and liver transplantation
Pontchaillou University Hospital
35033 Rennes Cedex 09, France
michel.rayar@chu-rennes.fr

Dr Fabien Robin
Department of HPB surgery and liver transplantation
Pontchaillou University Hospital
35033 Rennes Cedex 09, France
fabien.robin@chu-rennes.fr

Prof Emmanuel BOLESLAWSKI
Department of HPB surgery and liver transplantation
Claude Huriez University Hospital
Rue Michel Polonovski
59037 Lille Cedex, France
Phone: +33 3 20 44 42 60
emmanuel.boleslawski@chru-lille.fr

Prof Philippe BACHELLIER
Department of HPB surgery and liver transplantation
Hôpital Hautepierre
1 Avenue Molière
67200 Strasbourg, France

Phone: +3 33 88 12 72 58
philippe.bachellier@chru-strasbourg.fr

Prof Mircea CHIRICA
Department of HPB surgery and liver transplantation
Michallon University Hospital
Boulevard de la Chantourne
38700 La Tronche
Phone: +3 34 76 76 75 75
mchirica@chu-grenoble.fr

Dr Agnès BONADONA
Department of HPB surgery and liver transplantation
Michallon University Hospital
Boulevard de la Chantourne
38700 La Tronche
Phone: +3 34 76 76 62 57
abonadona@chu-grenoble.fr

Prof Olivier SCATTON
Department of HPB surgery and liver transplantation
Pitié Salpêtrière Hospital
83 Boulevard de l'hôpital
75013 Paris, France
Tel: +33 1 848 27427
olivier.scatton@aphp.fr
