# Supplementary material for: End-ischemic hypothermic oxygenated perfusion for extended criteria donors in liver transplantation: a multicenter, randomized controlled trial—HOPExt
Source: Trials. 2023 Jun 6;24:379. doi: 10.1186/s13063-023-07402-0 (PMC10243046; doi:10.1186/s13063-023-07402-0)
Supplement: Supplementary file 2 — Additional file 2. Health economic analysis. [file 13063_2023_7402_MOESM2_ESM.pdf]

1 HEALTH ECONOMIC ANALYSIS

1.1 Context of the medico-economic evaluation

To our knowledge, no study has been published on the efficiency of using HOPE in ECD grafts compared to static cold storage.

The hypothesis of the medico-economic evaluation is that HOPE could decrease 90-day morbidity and mortality and reduce the length of hospital stay and intermediate care unit stay. However, in a context with no published studies about the economic impact of the use of HOPE in ECD grafts, we propose to evaluate the efficiency of this procedure in comparison with the standard static cold storage.

Two strategies will be compared:

- the innovative strategy including the use of end-ischemic hypothermic oxygenated perfusion (HOPE) and,
- the standardized strategy with static cold storage.

1.2 Viewpoint

We will adopt the viewpoint of the French Public Health Insurance for the entire follow-up, with regard to the reimbursement of the health care. However, in order to highlight the cost of using HOPE for hospitals decision-makers, we will also value the procedure from the hospital point of view.

1.3 Time horizon

The time horizon from the French Public Health Insurance will be from the inclusion of the patient in the study until 12 months after. This time horizon will be sufficient to evaluate the whole costs of the clinical consequences for patients undergoing liver transplantation.

1.4 Costs of the HOPE procedure

We will first assess the cost of the HOPE using a micro-costing method to reconstitute the costs of performing a transplantation with this innovative technique, including the requirements for medical or paramedical personnel, needs in terms of specific consumables, and needs in terms of amortization and maintenance of the equipment.

Those costs will be valued using the following method:

- The cost of medical and non-medical staff involved in the procedure will be based on the annual number of full-time equivalents of each staff category, which will be estimated from charged harmonized gross salary means for the corresponding staff category. This annual staffing cost, in proportion to the annual capacity of the operating room, will allow us to obtain a staff cost per hour of activity of the operating room. The cost per patient in terms of operating room staff will be obtained by multiplying the duration of the occupation of the room (for every patient) by the hourly cost of the room. The cost of the interpretation of the results will be measured by the hourly cost of the medical staff multiplied by the time dedicated to the procedure. The data necessary for this calculation will be obtained from the Financial Services of every hospital participating in the study.

- The cost of the specific consumables (disposable kit, perfusion liquid) needed for each procedure will be obtained by multiplying the various quantities consumed for each patient in the study by the unit purchase price of every center.
- The annual maintenance cost for the equipment used during every procedure will be related to the annual hourly capacity of the operating room having the corresponding material in order to obtain an hourly maintenance cost (data obtained from the Financial Services). The maintenance cost for every patient in the study will be obtained from the product of the holding time of the operating room for every patient multiplied by the hourly maintenance cost.

The total cost will then be compared with the mean cost of the operating room of the Diagnosis Related Group 27C02 "Hepatic Transplantations" according to the National Study of Costs (ENC). This comparison will provide the information of the potential extra cost of the HOPE procedure compared with the standard static cold storage procedure.

1.5 Common costs to both strategies

We will then evaluate the costs of care for the 12 months following the intervention, from the point of view of the French Public Health Insurance. As patient care is essentially a hospital care, we will focus on hospital costs:

- Cost of hospital stays for the surgical procedure and re-hospitalizations for complications, based on current tariffs in the corresponding hospital stay related group (GHS).
- Costs of consultations based on the current tariffs of the general classification system for professional activities (NGAP),
- Costs of biological and radiological examinations based on the current tariffs from the national biology table (NABM) and the common classification of medical acts (CCAM).

1.6 Statistical analysis of costs

To the extent that the cost study will be conducted in parallel with the clinical study, the effects and costs will be obtained from sampled data from the same patients, and cost variables will not be analyzed as point estimates but stochastically, using conventional statistical methods. Cost data will be described in terms of means, variances and confidence intervals. The normality or non-normality of the distribution cost will be checked using the Kolmogorov-Smirnov test. If cost data are found to be non-normal, the standard non-parametric methods (such as the Mann-Whitney U test), the methods to bring the distribution costs closer to normality, or the bootstrap method will be used.

1.7 Incremental cost-effectiveness ratio

Once the mean cost and the mean effectiveness per patient are estimated for each of the two strategies, the economic evaluation will link the medical costs of each strategy with its consequences in physical units. The calculated incremental cost-effectiveness ratio (ICER) will indicate the additional costs due to the major adverse events that were avoided with the innovative strategy in comparison with the reference strategy.

$$ICER = \frac{\text{Cost of the innovative strategy} - \text{Cost of the reference strategy}}{\text{Effectiveness of the innovative strategy} - \text{Effectiveness of the reference strategy}}$$

1.8 Cost-effectiveness analysis

We will perform a cost-effectiveness analysis to obtain an incremental cost-effectiveness ratio, highlighting the extra cost per patient alive 12 months after transplantation with the innovative strategy compared to the usual strategy of patient care. To investigate the robustness of the results of the cost-effectiveness analysis, a sensitivity analysis will be carried out by changing some parameters of the study to measure the impact of the variation of these parameters on the final result.
