# Supplementary material for: End-ischemic hypothermic oxygenated perfusion for extended criteria donors in liver transplantation: a multicenter, randomized controlled trial—HOPExt
Source: Trials. 2023 Jun 6;24:379. doi: 10.1186/s13063-023-07402-0 (PMC10243046; doi:10.1186/s13063-023-07402-0)
Supplement: Supplementary file 3 — Additional file 3. Informed consent form. [file 13063_2023_7402_MOESM3_ESM.pdf]

Perfusion hypothermique oxygénée en fin d'ischémie des greffons à critères élargis en transplantation hépatique – essai randomisé multicentrique HOPExt

Note d'information à destination des patients
Version 5.0 du 27/12/2021

Promoteur :

Hospices Civils de Lyon
BP 2251
3 quai des Célestins,
69229 LYON cedex 02

Investigateur coordonnateur :

Prof Mickaël LESURTEL
Service de Chirurgie Hepatobiliaire
Hôpital Beaujon
100 Boulevard du General Leclerc, 92110 Clichy
Tél: +33 1 40 87 58 95
mickael.lesurtel@aphp.fr

Madame, Monsieur,

Votre médecin vous a présenté le protocole de recherche ***HOPExt*** *et sollicite votre accord pour que vous y participiez. Ce document a pour objectif de vous donner toutes les informations relatives à cette recherche de façon à vous permettre d'exercer au mieux votre liberté de décision. Ce document est obligatoire et son contenu est défini par le* ***Code de la Santé Publique, article L 1122-1*** *régissant les recherches impliquant la personne humaine.*

Il décrit précisément le protocole de recherche et mentionne toutes les autorisations réglementaires obtenues pour sa mise en œuvre.

Avant de prendre une décision, il est important que vous lisiez attentivement ces pages qui vous apporteront les informations nécessaires concernant les différents aspects de cette recherche. Vous devez conserver ce document. N'hésitez pas à poser des questions si vous ne comprenez pas certains éléments.

La signature du formulaire de consentement devenue obligatoire par l'application du code de la Santé Publique (livre I, titres 2 et 3 du CSP), n'affecte aucunement vos droits légaux.

Votre participation est entièrement volontaire. Si vous ne désirez pas prendre part à cette recherche, vous continuerez à bénéficier de la meilleure prise en charge médicale possible, conformément aux connaissances actuelles.

Pourquoi cette recherche?

L'organisation de la transplantation hépatique et l'attribution des greffons (foie du donneur) répondent à des règles précises d'attribution, gérées sur le plan national par l'agence de biomédecine.
Actuellement, il existe en France une pénurie de dons d'organes et donc de greffons hépatiques, ce qui a conduit la communauté médicale à proposer des greffons dit « à critères élargis » afin d'augmenter le nombre de greffons disponibles.
Un greffon « à critères élargis » correspond à un greffon dont les critères de sélection du donneur ont été élargis (par exemple l'âge du donneur, son poids …). Ces greffons dits « à critère élargis » sont plus sensibles au manque d'oxygène, qui peut se produire durant le transport dans le froid du greffon vers son receveur. Cela peut entrainer de plus fréquentes complications dans les suites immédiates de la transplantation.
Afin de remédier à ce problème et afin d'essayer d'améliorer la qualité de ces greffons, des machines de perfusion hépatique peuvent être utilisées. Cette technique, appelée HOPE (endischemic Hypothermic Oxygenated PErfusion) consiste à irriguer le greffon par un liquide de refroidissement très riche en oxygène. Cette étape se déroule au bloc opératoire pendant le retrait du foie malade du receveur et avant l'implantation du greffon. Cette technique a déjà fourni de bons résultats en transplantation du rein et du foie, sur un petit nombre de patients, et sur tous types de greffons hépatiques.

Vous êtes candidats à une transplantation hépatique, et vous êtes ou vous allez être inscrit(e) sur la liste nationale française d'attente de transplantation hépatique.
Nous vous proposons de participer à cette étude, qui va évaluer l'intérêt de la machine de perfusion hépatique par rapport à la prise en charge habituelle du greffon sur la survenue de complications précoces après la transplantation hépatique.

Quel est l'objectif de cette recherche?

Le but de cette recherche est donc **de vérifier l'intérêt d'une perfusion oxygénée hypothermique des greffons « à critères élargis » sur la récupération de fonction du greffon après transplantation par rapport à une conservation classique du greffon**.
Ce traitement innovant par machine de perfusion hépatique permet d'apporter des nutriments et de l'oxygène au greffon avant son implantation chez le receveur.
Par ailleurs, cette recherche a également pour objectif d'évaluer les coûts de prise en charge des patients ayant accepté de participer au projet afin de comparer les deux groupes (« machine de perfusion » et « conservation classique »). Dans ce cadre, des informations concernant vos consommations de ressource à l'hôpital (hospitalisations, consultations, actes) seront recueillies.

Quelle est la méthodologie de cette recherche ?

Il s'agit d'une recherche réalisée dans plusieurs centres français de transplantation hépatique équipés de la machine de perfusion HOPE. Deux groupes de patients seront comparés : le groupe « machine de perfusion » dans lequel le greffon sera connecté à la machine et le groupe « conservation classique » dans lequel le greffon sera conservé, comme habituellement, dans une solution statique hypothermique. Un total de 133 patients transplantés par groupe est nécessaire, soit 266 patients transplantés en tout.
Chaque patient participant à cette recherche se voit attribué à un des deux groupes par un tirage au sort. L'allocation à un des deux groupes se fait de manière aléatoire, **c'est la randomisation**. Ni le patient, ni le médecin référent ne peut choisir le groupe dans lequel le patient est alloué.
Si vous êtes dans le groupe « machine de perfusion », après son transport et sa préparation, le greffon prélevé sera perfusé par la solution froide et oxygénée avant l'implantation. Cette perfusion aura lieu en parallèle de votre préparation – pendant le retrait du foie malade – et ne retardera en aucun cas l'implantation du nouveau foie. De plus, la perfusion n'affectera en aucun cas la technique de transplantation.
Il est important de noter qu'il n'y a pas d'intervention directe sur votre corps puisque c'est uniquement le greffon qui est concerné par la perfusion ou non <u>avant l'implantation</u>.
Si vous êtes dans le groupe « conservation classique », après son transport et sa préparation, le greffon sera conservé dans une solution classique pendant votre préparation et le retrait de votre foie malade.

Comment va se dérouler cette recherche ?

L'étude vous sera présentée lors d'une visite avec le chirurgien, soit lors de votre inscription sur liste de transplantation ou soit lors d'une visite de suivi. Un temps de réflexion vous sera laissé. Une fois toutes vos questions posées et votre accord recueilli vous signerez avec le chirurgien un consentement.

Votre inclusion sera finalisée le jour de la transplantation. En effet si vous recevez un greffon à critères élargis vous serez randomisé dans l'étude, en revanche si vous ne recevez pas de greffon à critères élargis vous sortez de l'étude.

Votre suivi dans le cadre de cette étude sera de 1 an à partir de la transplantation hépatique.

La recherche peut se découper en plusieurs phases :
1. La transplantation hépatique pendant laquelle le greffon est perfusé ou non par la machine de perfusion en fonction du groupe dans lequel vous êtes alloué ;
2. La surveillance post-opératoire précoce en unité de soins intensifs pendant 1 semaine en moyenne, puis dans le service d'hospitalisation conventionnelle de chirurgie pendant 15 jours en moyenne ;

3. Après votre sortie de l'hôpital, une surveillance sera organisée conjointement par les services de chirurgie et d'hépatologie. Toutes les visites et examens sont réalisés dans le cadre de votre suivi habituel.

De nombreux examens vous seront faits pendant toute la durée de la recherche. **Ces examens sont réalisés dans la pratique courante, avant et après toute transplantation hépatique, que vous participiez ou non à la recherche**. Aucune prise de sang supplémentaire ne sera réalisée dans le cadre de la recherche. Le seul examen supplémentaire en rapport avec la recherche est une IRM hépatique réalisée 1 an après la transplantation juste avant la fin de votre participation à la recherche. L'objectif de cet IRM est de contrôler la qualité des voies biliaires qui auraient pu être altérées par le manque d'oxygène.

Au cours de cette recherche, en fonction de votre groupe, un ou deux prélèvements de foie supplémentaires seront réalisés sur le greffon afin de déterminer les dégradations liées au syndrome d'ischémie (manque d'oxygénation des organes). Pour le groupe « machine de perfusion », deux prélèvements seront réalisés sur le greffon : lors de sa préparation et après la perfusion. Pour le groupe « conservation classique », un seul prélèvement sera réalisé sur le greffon lors de sa préparation.

Les échantillons prélevés seront stockés à -80°C au sein de votre hôpital, pendant la durée de l'étude, puis envoyés au laboratoire I-Cube pour réaliser les analyses. Ce laboratoire est commun entre le CNRS (Centre national de la recherche scientifique) , l'Université de Strasbourg, l'ENGEES (École Nationale du Génie de l'Eau et de l'Environnement de Strasbourg) et de l'INSA (l'institut national des sciences appliquées) de Strasbourg et est situé au 300 Bd Sébastien Brant, 67400 Illkirch-Graffenstaden. Ces échantillons seront analysés dans le cadre de la recherche puis détruits conformément à la règlementation.

Aucun examen à caractère génétique n'est prévu au cours de cette recherche.

Quels sont les bénéfices et les contraintes liés à votre participation ?

En participant à cette recherche, vous contribuerez à l'amélioration des techniques de conservation des greffons et donc à l'optimisation de la transplantation hépatique.

Sur le plan individuel, si le greffon hépatique bénéficie de la perfusion par la machine sa qualité sera potentiellement optimisée avec une meilleure récupération de ses fonctions après la greffe.

Si vous acceptez de participer, vous devrez respecter les points suivants :

- Venir aux rendez-vous. En cas d'impossibilité, nous vous remercions de contacter votre médecin le plus rapidement possible.
- Informer le médecin responsable de la recherche de votre état de santé que ce soit pendant l'hospitalisation ou lors des rendez-vous après le retour à domicile.
- Informer le médecin responsable de la recherche de l'utilisation de tout médicament ainsi que de tout événement survenant pendant la recherche (hospitalisation, grossesse, …), y compris des traitements alternatifs par les plantes, les thérapies asiatiques et l'homéopathie.
- Ne pas prendre part à un autre projet de recherche avant la fin de votre suivi (environ 12 mois après la transplantation) sans l'accord de votre médecin, ceci pour vous protéger de tout accident possible pouvant résulter par exemple d'incompatibilités possibles entre traitements ou d'autres dangers.
- Être affilié(e) à un régime de sécurité sociale ou être bénéficiaire d'un tel régime.

Quels sont les risques prévisibles de la recherche ?

Il n'y a aucun risque supplémentaire à l'utilisation de la machine.

La solution de perfusion utilisée dans le cadre de la recherche est produite de manière synthétique, elle est stérile et n'est pas issue d'un organisme vivant. Ainsi, vous ne serez pas plus exposé au risque de transmission virale.

L'IRM n'est pas un examen douloureux, n'est pas une technique dite invasive et ne délivre aucun rayon X. Comme dans le cadre du soin, la réalisation de l'IRM 12 mois après votre transplantation peut induire de l'anxiété, une intolérance au bruit et de la claustrophobie.

D'autres effets sont prévisibles mais indépendants de la recherche puisque liés à la transplantation hépatique elle-même et aux traitements immunosuppresseurs.

Les effets secondaires liés à la transplantation hépatique sont :

- Pour les plus fréquents, non graves et attendus :
  - L'hypertension artérielle, l'insuffisance rénale ainsi qu'un risque d'infection. Ces effets secondaires sont connus et facilement prévenus par l'adaptation des médicaments et la prescription de façon systématique des vaccins.
  - Une fatigue anormale ou fatigabilité accentuée.
  - Ascite post-greffe (accumulation anormale de liquide dans l'abdomen), sténose biliaire (rétrécissement des voies biliaires) et infections bactériennes.
- Pour les plus rares et plus graves :
  - Une non fonction ou dysfonction du greffon qui serait potentiellement traitée par une retransplantation.
  - Des hémorragies post-opératoires qui seront traitées par transfusion et/ou ré-opération.
  - Des phénomènes de rejet du greffon qui seront traités efficacement de manière médicale.
  - Des fuites (fistules) biliaires qui seront traitées par drainage au contact du canal biliaire.

Quelles sont les éventuelles alternatives médicales ?

Votre refus de participer à la recherche n'influencera ni la prise en charge de votre maladie selon la procédure habituelle de transplantation hépatique, ni le délai d'attribution d'un nouveau foie. Les foies sont alloués nationalement selon la condition médicale du receveur et totalement indépendamment de cette recherche.
En dehors de cette recherche, le greffon qui vous aurait été alloué n'aurait pas été soumis à la perfusion.

Quels sont les traitements autorisés et non autorisés durant la recherche ?

Aucun traitement médicamenteux n'empêche la participation à la recherche.

Quels sont vos droits ?

- ***Participation volontaire***

Votre participation est volontaire ; vous êtes entièrement libre d'accepter ou de refuser de participer à cette recherche sans que cela ne modifie la qualité des soins auxquels vous avez droit, ou les relations existant avec votre médecin ou l'investigateur.

Si vous décidez de participer à cette recherche mais que vous changiez d'avis au cours de celle-ci, **vous pouvez à tout moment demander d'interrompre votre participation à la recherche sans aucun préjudice**, sans justification de votre part et sans que votre responsabilité ne soit engagée. Ce refus sera sans conséquence sur les soins qui seront alors prodigués conformément aux bonnes pratiques médicales en vigueur. Vos données recueillies jusque-là seront utilisées dans les résultats de la recherche.
D'autre part, s'il le juge nécessaire pour votre bien, l'investigateur pourra modifier votre suivi et vous pourrez continuer à bénéficier pleinement de sa compétence.

Plus généralement, votre participation à cette recherche ne décharge en aucune façon le promoteur et les investigateurs de leurs devoirs envers vous.

A tout moment, toutes les informations que vous souhaiteriez obtenir ultérieurement concernant cette recherche vous seront communiquées dans la mesure du possible par votre médecin et/ou par l'investigateur. Vous serez tenu informé de toute nouvelle donnée importante concernant cette recherche à laquelle vous acceptez de participer.
L'investigateur, tout comme le promoteur, peut interrompre à tout moment votre participation à la recherche s'il juge que cela est dans votre intérêt, ou arrêter la recherche dans sa globalité pour des raisons médicales, administratives ou autres.
Pour pouvoir participer à cette recherche, vous devez nécessairement être affilié à un régime d'assurance maladie telle que celui de la sécurité sociale.

Durant toute votre participation à cette recherche, il vous sera demandé de ne pas participer à une autre étude qui pourrait interférer avec les résultats du présent protocole de recherche.

Tous les frais médicaux liés à la recherche seront à la charge du promoteur. Il n'y aura pas de coût supplémentaire pour vous. Vous ne serez pas rémunéré(e) du fait de la participation à la recherche.

- ***Confidentialité et protection des données***

Dans le cadre de la recherche à laquelle les Hospices Civils de Lyon vous proposent de participer, un traitement informatique de vos données personnelles va être mis en œuvre pour permettre d'analyser les résultats de la recherche au regard de l'objectif de cette dernière. Le responsable du traitement des données est le promoteur, dont les coordonnées figurent sur la première page de ce document. Ce traitement des données a pour fondement juridique l'article 6 du Règlement Général sur la Protection des Données (RGPD) à savoir l'exécution d'une mission d'intérêt public dont est investi le responsable de traitement et les intérêts légitimes poursuivis par lui. De plus, au titre de l'article 9 du RGPD le responsable de traitement peut de manière exceptionnelle traiter des catégories particulières de données, incluant des données de santé notamment à des fins de recherche scientifique.

Pour l'analyse, les données médicales vous concernant seront transmises aux Hospices Civils de Lyon ou aux personnes ou sociétés agissant pour son compte en France ou à l'étranger. En cas de transfert de données à caractère personnel hors de l'Union Européenne et/ ou vers un pays ne garantissant pas un niveau de protection suffisant par rapport à l'Union Européenne ou à une organisation internationale, le promoteur et/ou le responsable de traitement mettront en place des garanties appropriées pour ce transfert (Clauses Contractuelles Spécifiques). Si vous souhaitez obtenir une copie des Clauses Contractuelles Spécifiques, vous pouvez vous adresser au Délégué à la Protection des Données (DPO) du promoteur à l'adresse suivante : **dpo@chu-lyon.fr.** Ces données seront identifiées par un code et vos initiales. Ces données pourront également, dans des conditions assurant leur confidentialité, être transmises aux autorités de santé françaises ou étrangères et à d'autres entités en dehors des Hospices Civils de Lyon, notamment au laboratoire I-Cube, partenaire des HCL pour cette étude.

Les données seront transférées et collectées conformément à la méthodologie de référence MR001 de la Commission Nationale de l'Informatique et des Libertés (CNIL) pour laquelle les Hospices Civils de Lyon ont signé un engagement de conformité. Conformément à la réglementation française et européenne, les données de la recherche seront conservées 25 ans.

Avec votre accord, votre médecin traitant sera informé de votre participation à la recherche.
Par ailleurs, sauf opposition expresse de votre part adressée à l'investigateur coordonnateur dont les coordonnées figurent sur la première page de ce document, vos données recueillies dans le cadre de cette recherche pourront être transmises ailleurs dans le monde et réutilisées par des partenaires publics ou privés lors de recherches ultérieures exclusivement à des fins scientifiques.

Si vous avez des questions ou des réclamations au sujet du traitement de vos données au cours de cette recherche, vous pouvez contacter le DPO par voie électronique **: dpo@chu-lyon.fr** ou par courrier postal :
Le délégué à la protection des données
162 avenue Lacassagne
Bâtiment A – 3e étage – Bureau 316
69003 LYON
Si vous estimez, après avoir contacté le DPO des HCL, que vos droits sur vos données ne sont pas respectés, vous pouvez adresser une réclamation (plainte) à la CNIL :
https://www.cnil.fr/fr/webform/adresser-une-plainte

- ***Exercer vos droits***

Vous pourrez également, à tout moment, exercer votre droit d'accès, de vérification, de correction, de limitation et d'opposition au traitement et à la transmission des données vous concernant en en faisant la demande auprès du médecin de votre choix ou auprès d'un investigateur de la recherche. Si vous souhaitez exercer votre droit à l'effacement de vos données, le responsable de traitement peut au titre des Articles 17.3.c et 17.3.d. du RGPD ne pas faire droit à cette demande si celle-ci est susceptible de rendre impossible ou de compromettre gravement la

réalisation des objectifs de la recherche. Ainsi, vos données recueillies préalablement au retrait de votre consentement pourront ne pas être effacées et pourront continuer à être traitées dans les conditions prévues par la recherche.

Si les résultats de cette recherche devaient être présentés dans des communications et/ou des publications scientifiques médicales, l'identité des participants n'apparaîtra d'aucune façon.

A l'issue de cette recherche, les résultats globaux pourront vous être communiqués sur simple demande auprès de l'investigateur coordonnateur, le Pr Mickaël LESURTEL. La base de données de cette recherche rendue totalement anonyme pourra être transmise à d'autres chercheurs qui travailleraient sur le même sujet.

- ***Dispositions réglementaires***

La recherche sera conduite conformément aux lignes directives des Bonnes Pratiques Cliniques françaises et européennes, à la déclaration d'Helsinki dans sa dernière version, aux recommandations de l'ICH (International Conference on Harmonisation), Guideline for Good Clinical Practice ainsi qu'aux dispositions législatives et réglementaires en vigueur.
Le Comité de Protection des Personnes Ile de France III (Hôpital Tarnier-Cochin – 89 rue d'Assas – 75003 Paris) a émis un avis favorable à la réalisation de cette recherche le 05/06/2019. L'Agence nationale de sécurité du médicament et des produits de santé (ANSM) a également donné son autorisation à la mise en œuvre de cette recherche le 20/06/2019. Enfin, cette recherche respecte le règlement général sur la protection des données.
Le promoteur de cette recherche, les Hospices Civils de Lyon, BP 2251, quai des célestins, 69229 Lyon cedex 02, a souscrit une assurance de responsabilité civile auprès de la Société Hospitalière d'Assurance Mutuelle, 18 rue Edouard Rochet, 69008 Lyon, sous le numéro 159.077.
Les personnes ayant subi un préjudice après participation à une recherche interventionnelle peuvent faire valoir leurs droits auprès de l'assureur du promoteur.

L'investigateur doit vous fournir toutes les explications nécessaires concernant cette recherche. Vous avez le droit d'arrêter votre participation à quelque moment que ce soit, et quel que soit le motif ; vous continueriez à bénéficier du suivi médical et cela n'affectera en rien votre surveillance future.

Qui pouvez-vous contacter pour toute question ?

Si vous avez des questions concernant cette recherche, n'hésitez pas à nous les poser. Nous pouvons vous donner les informations complémentaires que vous souhaitez. Les noms et numéros de téléphone des personnes à contacter sont les suivants :

Investigateur coordonnateur de la recherche :
Service de Chirurgie Hepatobiliaire
Hôpital Beaujon
100 Boulevard du General Leclerc
92110 Clichy
Tél: +33 1 40 87 58 95
mickael.lesurtel@aphp.fr
Investigateur de votre centre référent pour la recherche :
……………………………….
……………………………….
……………………………….
……………………………….
……………………………….

Nous vous remercions de l'attention que vous avez portée à la lecture de cette notice. Une copie de ce document vous sera remise pour que vous puissiez bénéficier de l'ensemble des informations concernant votre participation à cette recherche.

Lorsque vous aurez lu cette note d'information, il vous sera proposé, si vous êtes d'accord, de donner votre consentement écrit en signant le formulaire préparé à cet effet.

Hospices Civils de Lyon<br>**Direction de la Recherche en Santé**	**FORMULAIRE DE CONSENTEMENT A L'ATTENTION DES PATIENTS**

La loi 2012-300 du 5 mars 2012 relative aux recherches impliquant la personne humaine rend obligatoire le recueil de l'accord écrit des patients sollicités pour participer à toute recherche interventionnelle ou recherche interventionnelle à risques et contraintes minimes. C'est un tel accord qui vous est demandé ci-dessous, pour participer à la recherche intitulée :

Perfusion hypothermique oxygénée en fin d'ischémie des greffons à critères élargis en transplantation hépatique – essai randomisé multicentrique
HOPExt

Promoteur : Hospices Civils de Lyon
BP 2251
3 quai des Célestins,
69229 LYON cedex 02

Investigateur coordonnateur : Prof Mickaël LESURTEL
Service de Chirurgie Hepatobiliaire
Hôpital Beaujon
100 Boulevard du General Leclerc, 92110 Clichy
Tél: +33 1 40 87 58 95
mickael.lesurtel@aphp.fr

Je soussigné(e) *(nom, prénom)* …………………………………………………………………….certifie avoir lu et compris la note d'information qui m'a été remise.

J'ai eu la possibilité de poser toutes les questions que je souhaitais au *Pr/Dr*.. *(nom, prénom)* qui m'a expliqué la nature, les objectifs, les risques potentiels et les contraintes liées à ma participation à cette recherche.

Je connais la possibilité qui m'est réservée d'interrompre ma participation à cette recherche à tout moment sans avoir à justifier ma décision et je ferai mon possible pour en informer l'investigateur qui me suit dans la recherche. Cela ne remettra naturellement pas en cause la qualité des soins ultérieurs.

J'ai eu l'assurance que les décisions qui s'imposent pour ma santé seront prises à tout moment, conformément à l'état actuel des connaissances médicales.

J'ai bien compris que l'investigateur peut interrompre à tout moment ma participation à l'essai s'il le juge nécessaire.

Je suis informé(e) que mes données recueillies dans le cadre de cette recherche peuvent être transférées à des tiers et notamment au laboratoire I Cube (échantillons) pour analyses.

Je suis informé(e) de la possibilité que mes données recueillies dans le cadre de cette recherche puissent être réutilisées lors de recherches ultérieures exclusivement à des fins scientifiques et que je peux m'y opposer.

J'ai bien noté / été informé que cette recherche a reçu l'avis favorable du Comité de Protection des Personnes Ile de France III le 05/06/2019 et l'autorisation de l'ANSM le 20/06/2019 et a fait l'objet d'une déclaration à la Commission Nationale Informatique et Libertés (CNIL).

J'ai bien noté que cette recherche est menée conformément aux articles L1121-1 et suivants du Code de la Santé Publique, relatifs à la protection des personnes qui se prêtent à des recherches impliquant la personne humaine et conformément à la règlementation en vigueur.

Je certifie sur l'honneur être affilié à un régime de sécurité sociale ou bénéficiaire d'un tel régime.

Le promoteur de la recherche, les Hospices civils de Lyon, BP 2251, quai des célestins, 69229 Lyon cedex 02 a souscrit une assurance de responsabilité civile en cas de préjudice auprès de de la Société Hospitalière d'Assurance Mutuelle, 18 rue Edouard Rochet, 69008 Lyon, sous le numéro 159.077.

J'accepte que les personnes qui collaborent à cette recherche ou qui sont mandatées par le promoteur, ainsi qu'éventuellement le représentant des Autorités de Santé, aient accès à l'information contenue dans mon dossier médical dans le respect le plus strict de la confidentialité.

J'accepte que les données enregistrées à l'occasion de cette recherche puissent faire l'objet d'un traitement informatisé sous la responsabilité du promoteur.

J'ai bien noté que, conformément aux dispositions de la loi relative à l'informatique, aux fichiers et aux libertés, je dispose d'un droit d'accès, de rectification, de vérification, de correction et d'opposition à la transmission de mes données couvertes par le secret professionnel susceptibles d'être utilisées dans le cadre de cette recherche et d'être traitées. Ces droits s'exercent auprès de l'investigateur qui me suit dans le cadre de cette recherche et qui connaît mon identité.

J'ai été avisé qu'aucune indemnité n'est prévue pour ma participation à cette recherche.

Mon consentement ne décharge en rien l'investigateur et le promoteur de la recherche de leurs responsabilités à mon égard. Je conserve tous les droits garantis par la loi.

Les résultats globaux de la recherche me seront communiqués directement si j'en fais la demande, conformément à la loi du 4 mars 2002 relative aux droits des malades et à la qualité du système de santé.
Je peux à tout moment demander des informations complémentaires au Dr/Pr …………………………………………..

Deux exemplaires originaux de ce formulaire de consentement ont été établis : un m'a été remis, l'autre a été remis à l'investigateur et sera conservé au minimum 25 ans après la fin de la recherche.

- **Volontaire ou patient donnant son consentement :**

Ayant disposé d'un temps de réflexion suffisant avant de prendre ma décision, j'accepte librement et volontairement de participer à la recherche *HOPExt.*

NOM, Prénom du volontaire ou patient participant à la recherche :
……………………………………………………………………………………………………

Fait à : ……………………………………………………, le |___|___| / |___|___| / |___|___|___|___|

Signature du volontaire/patient :

- **Investigateur obtenant le consentement :**

J'atteste que toutes les obligations liées à un consentement éclairé ont été satisfaites dans le cadre de ce projet de recherche clinique – que le participant a reçu une information relative à ses droits, que nous avons discuté de ce projet et que je lui ai expliqué en termes compréhensibles l'ensemble des informations contenues dans la notice. Je certifie également avoir laissé le participant me poser toutes les questions qu'il souhaitait et y avoir répondu.

NOM, Prénom de l'investigateur : ………………………………………………………………………………………………….

Fait à : ……………………………………………………, le |___|___| / |___|___| / |___|___|___|___|

Signature de l'investigateur :
